# Supplementary material for: Gephyrin-Lacking PV Synapses on Neocortical Pyramidal Neurons
Source: Int J Mol Sci. 2021 Sep 17;22(18):10032. doi: 10.3390/ijms221810032 (PMC8467468; doi:10.3390/ijms221810032)
Supplement: Supplementary file 1 [file ijms-22-10032-s001.zip › ijms-1342237-supplementary.pdf]

International Journal of
Molecular Sciences

MDPI

Supplemental Materials

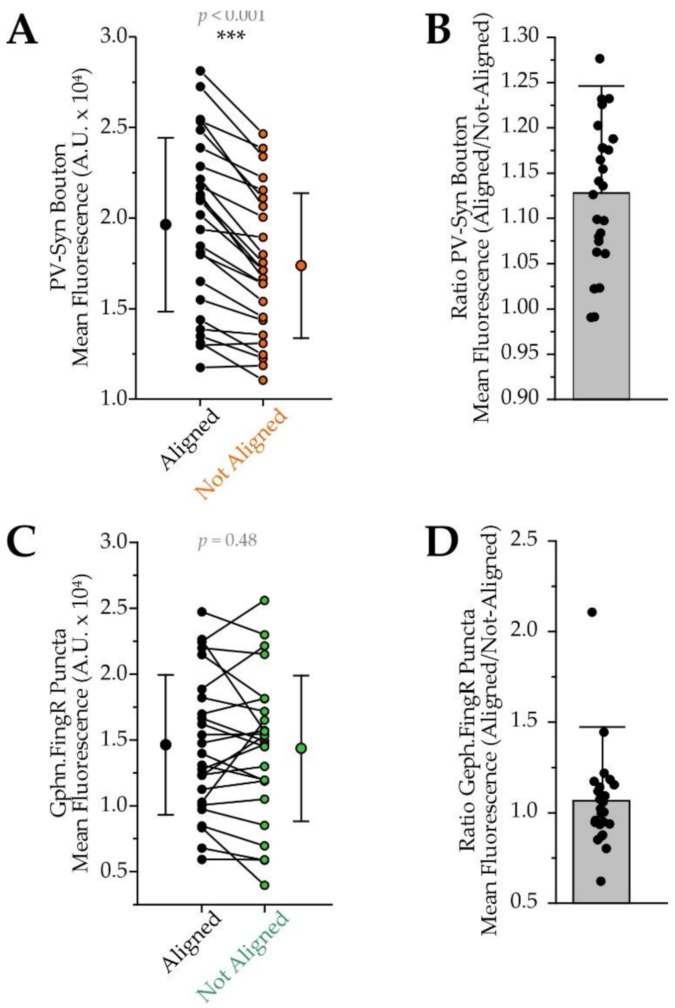

Figure S1. Fluorescence intensity of PV boutons and gephyrin puncta across Pyr neurons by alignment category; (**A**) PV-Syn bouton mean fluorescence intensity for boutons aligned with postsynaptic Gephryin.FingR (black) or not aligned (orange). Pairs of dots connected by a line represent the values for one Pyr neuron. Beside cell values are plotted means ± SD for each alignment category; (**B**) the ratio of mean PV-Syn bouton fluorescence intensity for the median bouton for each Pyr cell that could be aligned with Gephyrin.FingR over not aligned; (**C**) Gephyrin.FingR puncta (Gphn.FingR) mean fluorescence intensity for puncta aligned with presynaptic PV-Syn (black) or not aligned (green). Pairs of dots connected by a line represent the values for one Pyr neuron. Beside cell values are plotted means ± SD for each alignment category; (**D**) the ratio of mean Gephyrin.FingR fluorescence intensity for the median puncta for each Pyr cell that could be aligned with PV-Syn over not aligned; L2/3, n =13 cells; L5, n = 12 cells; N = 2 animals.

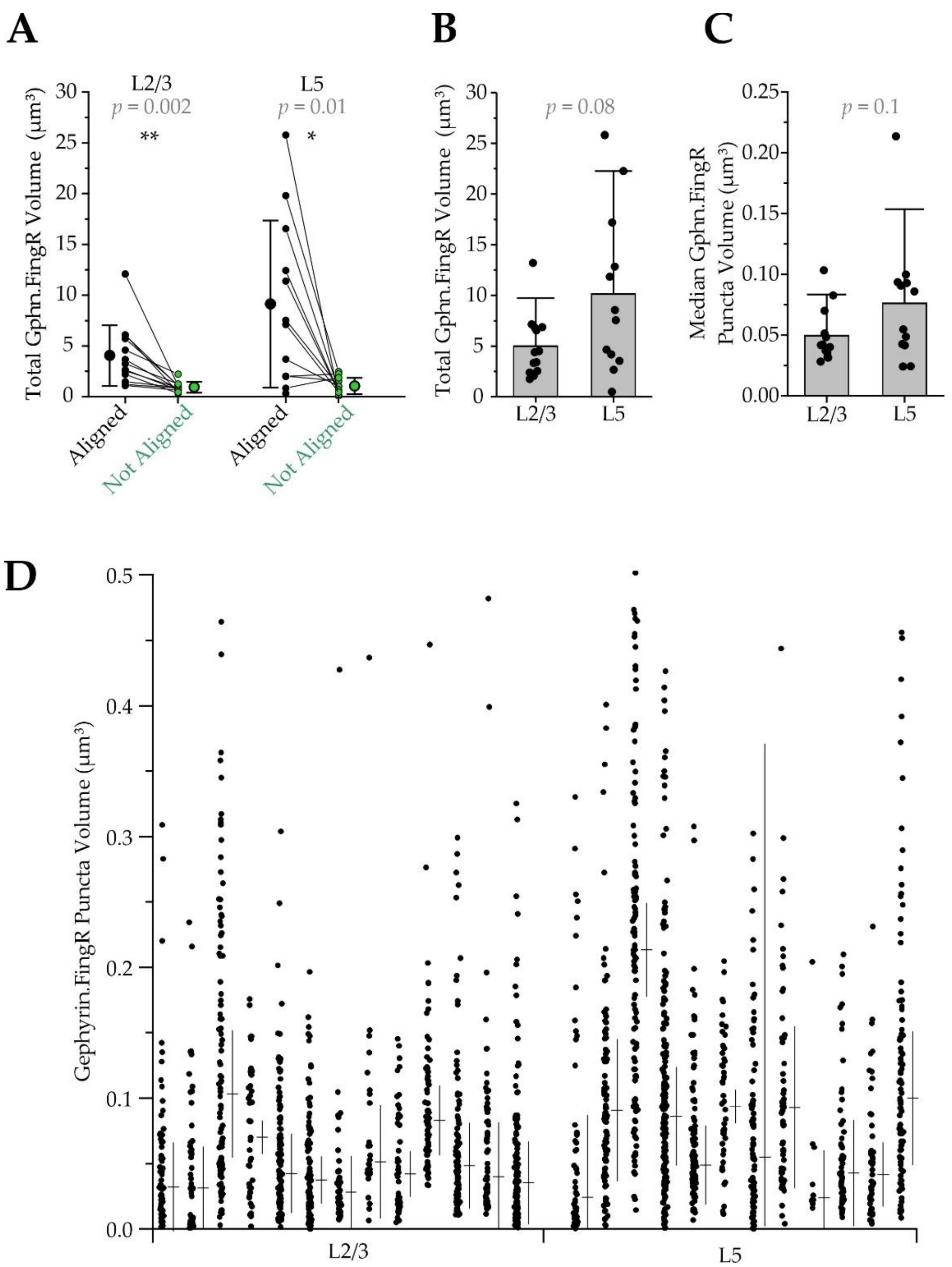

Figure S2. Total gephyrin volume and median gephyrin puncta sizes are similar for L2/3 and L5 Pyr neurons; (**A**) total Geghyrin.FingR puncta volume for L2/3 and L5 Pyr neuron somas based on whether puncta are aligned with presynaptic PV-Syn (black) or not aligned (green). Pairs of dots connected by a line represent the values for one Pyr neuron. Beside individual cell values are plotted means ± SD for each alignment category; (**B**) total Geghyrin.FingR puncta volume for L2/3 and L5 Pyr neuron somas; (**C**) median Gephyrin.FingR puncta volume for L2/3 and L5 Pyr neuron somas; (**D**) all Gephyrin.FingR puncta volumes for individual L2/3 and L5 Pyr neuron somas, median ± 95% confidence interval plotted to the right of each cell's data; L2/3, n =13 cells; L5, n = 12 cells; N = 2 animals.
